# Specific detection of interferon regulatory factor 5 (IRF5): A case of antibody inequality

**DOI:** 10.1038/srep31002

**Published:** 2016-08-02

**Authors:** Dan Li, Saurav De, Dan Li, Su Song, Bharati Matta, Betsy J. Barnes

**Affiliations:** 1 Center for Autoimmune and Musculoskeletal Diseases, Feinstein Institute for Medical Research, North well Health, Manhasset, NY 11030, United States; 2 Rutgers Biomedical and Health Sciences, New Jersey Medical School-Cancer Center, Newark, NJ 07103, United States; 3 Department of Microbiology, Biochemistry & Molecular Genetics, Rutgers Biomedical and Health Sciences, Newark, NJ 07103, United States.

## Abstract

Interferon regulatory factor 5 (IRF5) is a member of the IRF family of transcription factors. IRF5 was first identified and characterized as a transcriptional regulator of type I interferon expression after virus infection. In addition to its critical role(s) in the regulation and development of host immunity, subsequent studies revealed important roles for IRF5 in autoimmunity, cancer, obesity, pain, cardiovascular disease, and metabolism. Based on these important disease-related findings, a large number of commercial antibodies have become available to study the expression and function of IRF5. Here we validate a number of these antibodies for the detection of IRF5 by immunoblot, flow cytometry, and immunofluorescence or immunohistochemistry using well-established positive and negative controls. Somewhat surprising, the majority of commercial antibodies tested were unable to specifically recognize human or mouse IRF5. We present data on antibodies that do specifically recognize human or mouse IRF5 in a particular application. These findings reiterate the importance of proper controls and molecular weight standards for the analysis of protein expression. Given that dysregulated IRF5 expression has been implicated in the pathogenesis of numerous diseases, including autoimmune and cancer, results indicate that caution should be used in the evaluation and interpretation of IRF5 expression analysis.

The interferon regulatory factor 5 (*IRF5*) gene encodes proteins that bind to DNA and regulate the transcription of genes important for innate and adaptive immunity, as well as cell growth regulation and apoptosis^1^,^2^,^3^,^4^,^5^,^6^,^7^. *IRF5* was originally cloned from an expressed sequence tag (EST) human dendritic cell cDNA library and human B cells^1^. The first analysis of its expression was by Northern blot using a commercially available human healthy donor tissues blot. Constitutive *IRF5* mRNA expression was detected primarily in lymphoid tissues and peripheral blood lymphocytes (PBL) with the highest levels detected in spleen and PBL^1^. In all samples identified to express *IRF5*, two transcripts were detected. Subsequent findings from multiple labs have now shown that IRF5 exists as multiple alternatively spliced transcripts that encode distinct isoforms^3^. *IRF5* expression was also analyzed in human tumor cell lines and those cell lines expressing *IRF5* showed only one transcript^1^,^3^. Notably, all immortalized lymphoma and leukemia cell lines examined at that time showed the absence of *IRF5* mRNA expression.

To date, IRF5 is best known for its 1) central role in pathogen-induced immunity via activation by the MyD88-dependent Toll-like receptor (TLR) signaling pathway^8^,^9^, 2) identification as an autoimmune susceptibility gene^10^,^11^,^12^,^13^,^14^,^15^,^16^,^17^,^18^,^19^, and 3) tumor suppressor function^4^,^6^,^7^,^20^,^21^. With the availability of *Irf5* transgenic knockout mice, however, IRF5 has been also shown to play important roles in macrophage polarization, pain management, obesity, cardiovascular disease, systemic lupus erythematosus (SLE), arthritis and metabolic dysfunction^19^,^22^,^23^,^24^,^25^,^26^,^27^,^28^,^29^,^30^,^31^. IRF5 proteins generally reside in the cytoplasm of a quiescent cell and upon stimulation (with virus, TLR ligands or DNA damage, etc.) become activated via post-translational modification(s), resulting in nuclear translocation and binding to the promoters of target genes^2^. IRF5’s function (or dysfunction) in autoimmune disease and cancer is tightly linked to its expression. In the case of autoimmune diseases such as SLE, polymorphisms in the *IRF5* gene, referred to as risk polymorphisms, result in, or associate with, elevated IRF5 expression^11^,^32^,^33^,^34^. Conversely, in the case of cancer, IRF5 expression is often found to be downregulated (or absent) in malignant versus non-malignant cells. More recent data on the analysis of IRF5 expression and function in different cancer types has revealed that in some cases, such as in splenic marginal zone lymphoma, IRF5 expression is decreased^21^, while in Hodgkin’s lymphoma, IRF5 expression is elevated^35^. The same may be true for autoimmune diseases as both risk and non-risk polymorphisms have been associated with susceptibility to and protection from, respectively, a particular disease. In either case, analysis of IRF5 expression by immune-based techniques, such as immnuoblot, flow cytometry, and immunohistochemistry, has become very important for our understanding of IRF5 function (or dysfunction) in a disease-specific context.

In the current study, we used established positive and negative controls for the analysis of IRF5 antibody specificity in human cells and tissues, and *Irf5* gene knockout (ko) mice for analysis in murine cells and tissues. Data presented herein indicate that caution should be used in the interpretation of findings from particular antibodies as many are not specific for IRF5; a number of commonly used antibodies detect IRF5 in negative controls. We have identified a few key antibodies that are very good at the specific detection of IRF5 expression in particular applications. Given the clinical utility of IRF5 expression as a biomarker of disease susceptibility and severity, appropriate controls and molecular weight standards should be included and published in order to authenticate antibody specificity, as well as scientific findings.

## Results

**Figure 1:**
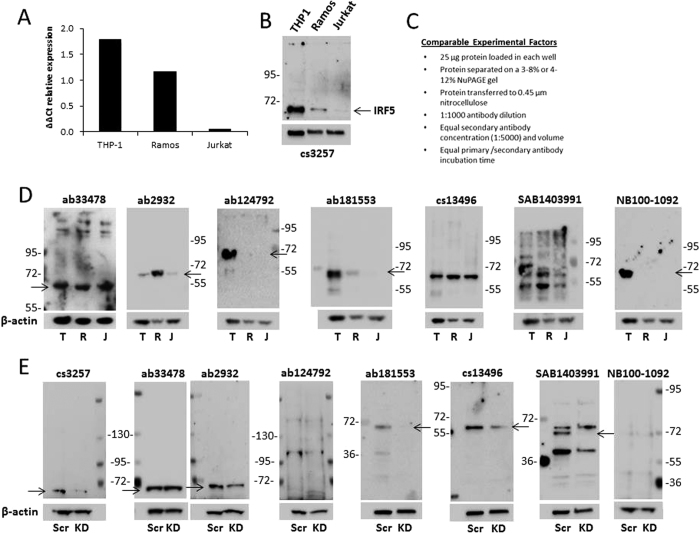
Comparative analysis of IRF5 protein expression between immortalized lymphoid cell lines by immunoblot. (**A**) qRT-PCR analysis of *IRF5* transcript expression in immortalized lymphoid cell lines. Data is presented as relative expression after normalization to β-actin using the ∆∆Ct method. (**B**) Immunoblot analysis of endogenous IRF5 protein expression in immortalized cell lines. IRF5 expression was detected with the previously validated Cell Signaling antibody #3257 that is no longer available. β-actin levels and apparent molecular weight standards are shown as loading controls and for size comparison, respectively. (**C**) Experimental details for the comparative analysis of α-IRF5 antibody specificity by immunoblot analysis. (**D**) Same as in (**B**)except lysates (T, THP-1; R, Ramos B cells; J, Jurkat T cells) were run on multiple independent blots for the analysis of antibody specificity; seven different commercially available α-IRF5 antibodies were evaluated. (**E**) Same as in (**D**) except antibodies were evaluated by IRF5 knockdown analysis. Ramos B cells were nucleofected with scrambled (Scr) or *IRF5* (KD) siRNAs and lysates from same nucleofection run on multiple independent blots for comparative analysis. Arrows indicate detection of an appropriately sized band(s) corresponding to IRF5. Data (except in (**C**)) are representative of three independent experiments.

In primary leukocytes and lymphocytes, IRF5 expression is highest in monocytes and macrophages, lower in dendritic cells and B cells, and nearly undetectable in T cells and NK cells. For the validation of currently available commercial anti-IRF5 antibodies, three immortalized human lymphoid cell lines – THP1 monocytic macrophages, Ramos B cells, and Jurkat T cells – that express distinctly different levels of endogenous IRF5 transcript and protein expression were used. Expression differences were confirmed by real-time quantitative polymerase chain reaction (qRT-PCR) and immunoblot analysis using lysates from cell lines newly obtained from ATCC and commercially purchased lysates (Fig. 1A,B). Protein levels were confirmed with previously validated antibodies that are no longer available - Cell Signaling #3257 (cs3257) or clone 2E3-1A11 from Novus Biologicals (H00003663-M01) and Sigma (WH0003663M1). As expected, cs3257 detected high levels of IRF5 in protein lysates from THP1 cells^1^,^3^. Compared to THP1, Ramos B cells expressed dramatically lower levels, and Jurkat T cells, as most T cell lines, expressed little to no IRF5 (Fig. 1B). Surprisingly, we have found that some companies used Jurkat T cells as a positive control for IRF5 protein detection; to our knowledge, there have been no publications showing IRF5 expression in Jurkat cells. To the contrary, two recent publications have shown that Jurkat T cells lack IRF5 expression^36^,^37^. Furthermore, analysis of Jurkat RNAseq reads from ENCODE using the UCSC Genome Browser indicate that Jurkat cells either express too low *IRF5* that reads are not detected across exons or expression is absent^38^,^39^.

Using protein lysates from the three cell lines, seven commercially available anti-IRF5 antibodies were tested for detection specificity by comparing to the previously available and validated cs3257 antibody. Key experimental factors, protein loading, antibody concentration, etc., were controlled for each antibody and are detailed in Fig. 1C. Although the majority of antibodies tested were able to detect a band (or bands) between the 72 and 55 kDa protein markers that correspond to the approximate size of endogenous IRF5 (~62 kDa), most antibodies were unable to distinguish IRF5 expression differences between cell lines (Fig. 1D). Antibodies #33478 (Abcam; ab33478) and #13496 (Cell Signaling; cs13496) detected similar, high IRF5 expression in all three cell lines indicating lack of specificity. Antibodies #124792 (Abcam; ab124792) and NB100-1092 (Novus Biologicals) detected a similar large, single band in THP1 cells but were unable to detect IRF5 in Ramos B cells; a faint band of appropriate size could be detected in lysates from Ramos B cells after longer exposures with ab124792 but not NB100-1092. While antibody #2932 (Abcam; ab2932) detected clean bands of appropriate size in all three cell lines, the levels of expression were incorrect showing Ramos B cells as the highest expressers of IRF5. Antibody SAB1403991 (Sigma) was the least specific as it detected multiple bands throughout the membrane and antibody #181533 (Abcam; ab181533) was the most specific since it cleanly detected expression differences between the cell lines. Ab181533 was the only antibody capable of detecting IRF5 expression differences between each cell line that correlated with transcript levels and detection with cs3257.

To further confirm antibody specificity, targeted siRNAs were used to knockdown endogenous IRF5 in Ramos B cells. qRT-PCR and immunoblot analysis with the validated cs3257 antibody were used to confirm IRF5 knockdown (Fig. 1E and data not shown). Lysates from the same batch of nucleofected cells were used to generate multiple independent blots for comparison of antibody specificity. Similar to immunoblot data in panel D, antibodies ab33478 and ab2932 were unable to detect differences in IRF5 expression after knockdown, and ab124792 and NB100-1092 were unable to detect IRF5 in Ramos B cells at all. Interestingly, although multiple bands were detected with SAB1403991, one band of appropriate size disappeared after knockdown suggesting that the antibody may be able to recognize IRF5, albeit in a non-specific manner. Antibody cs13496 was also interesting as it detected IRF5 knockdown in Ramos B cells even though it was unable to distinguish expression differences between the three cell lines (Fig. 1D). However, the most specific antibody for IRF5 detection, before and after knockdown, was ab181533 (Fig. 1E). Ab181533 was the most consistent and specific in its ability to detect differing levels of endogenous IRF5 by immunoblot analysis.

**Figure 2:**
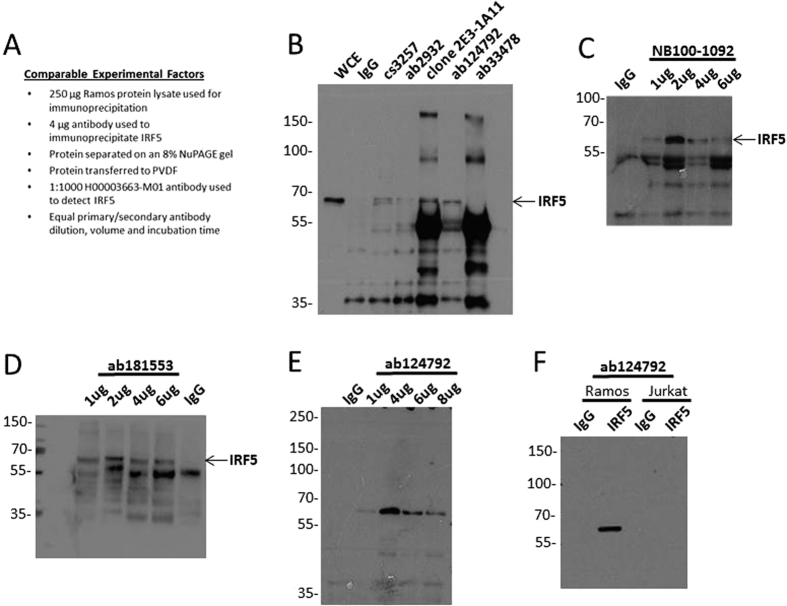
Ability of α-IRF5 antibodies to immunoprecipitate endogenous IRF5 from Ramos B cells. (**A**) Experimental details for the comparative analysis of α-IRF5 antibody specificity by immunoprecipitation. (**B–E**) Lysates from Ramos B cells were immunoprecipitated with the indicated antibodies and IRF5 detected by immunoblot. In (**C–E**), the indicated antibodies were titrated for immunoprecipitation. (**F**) Same as (**B**) except lysates from Jurkat T cells were also immunoprecipitated to test antibody specificity. Arrows indicate detection of an appropriately sized band(s) corresponding to IRF5. Data are representative of three independent experiments.

We next evaluated the ability and efficiency of IRF5 antibodies to immunoprecipitate (IP) endogenous IRF5 proteins from Ramos B cell lysates. Even though antibodies NB100-1092 and ab2932 were not able to recognize IRF5 by immunoblot analysis, we tested them for IP as the companies specifically recommended them for this method on their technical sheets. Cs3257 and clone 2E3-1A11 (WH0003663M1 or H00003663-M01) were also used for IP comparison as antibodies from clone 2E3-1A111 were the only ones previously available that could IP endogenous IRF5. Protein lysates were immunoprecipitated with the indicated antibodies, as detailed in Fig. 2A, and IP efficiency assessed by immunoblot analysis with H00003663-M01. Interestingly, cs3257 was unable to IP much IRF5 while WH0003663M1 (clone 2E3-1A11) could. Given that WH0003663M1 and H00003663-M01 were both monoclonal, high background bands for heavy and light chain immunoglobulins were seen on the blot, in addition to a band corresponding to IRF5 (Fig. 2B). Not surprising, ab33478 and ab2932 were unable to IP IRF5, while ab124792 immunoprecipitated similar levels as the previously available WH0003663M1, yet with less non-specific binding. Since NB100-1092 was recommended by the manufacturer for IP, we performed an antibody titration to detect IP efficiency and specificity. Somewhat surprising given the immunoblot data in Fig. 1D,E, NB100-1092 was able to precipitate large amounts of IRF5 with 2 μg of antibody; however, a number of other proteins precipitated with IRF5 indicating a lack of specificity (Fig. 2C). Ab181553 was also tested, given its sensitivity and selectivity for IRF5 by WB, and while it was able to IP IRF5, additional non-specific bands were detected (Fig. 2D). Since ab124792 gave the least non-specific binding (Fig. 2B), this antibody was further optimized by titration and washing after IP. We found highly efficient and specific IP of IRF5 at 4 μg antibody (Fig. 2E). To further confirm specificity, ab124792 was used for IP in Jurkat T cells and no binding was detected at the same concentration of protein lysate and antibody used for IP of Ramos B cell lysates (Fig. 2F). Of interest, Abcam specifically states on their product sheet that ab124792 is unsuitable for IP. Based on the current analysis, we found ab124792 to be a specific and efficient antibody for IP of human IRF5.

**Figure 3:**
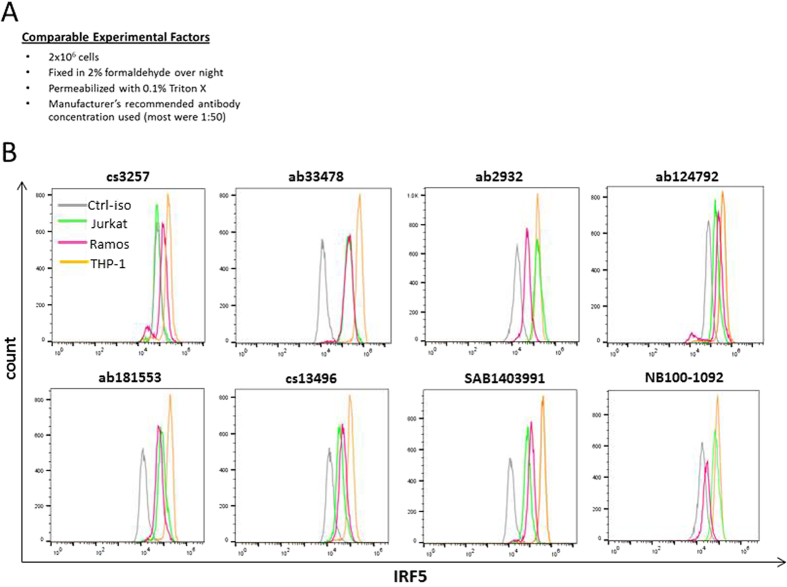
Comparative analysis of IRF5 protein expression between immortalized lymphoid cell lines using flow cytometry. (**A**) Experimental details for the comparative analysis of α-IRF5 antibody specificity by flow cytometry. (**B**) Representative histogram plots from flow cytometry analysis of intracellular IRF5 in immortalized cell lines using different α-IRF5 antibodies. Data are representative of three independent experiments. Ctrl-iso, matched isotype antibodies used as non-specific controls.

Next, to determine whether any of the antibodies were capable of detecting intracellular IRF5 by flow cytometry analysis, as in Fig. 1, IRF5 expression was compared between THP1, Ramos, and Jurkat cells. Using the same antibody concentration, permeabilization protocol, and cell staining procedure (Fig. 3A), we found that antibodies cs3257, ab124792, cs13496, and SAB1403991 were able to detect the expected IRF5 expression differences between cell lines (Fig. 3B). Ab124792 was specifically recommended for flow cytometry on its technical sheet and it showed the largest difference between cell peaks (based on MFI values). While not used in the current study, ab124792 is now available in pre-conjugated forms (ab193245 or ab192983) that we have found to further enhance specificity for intracellular IRF5 detection by flow cytometry.

**Figure 4:**
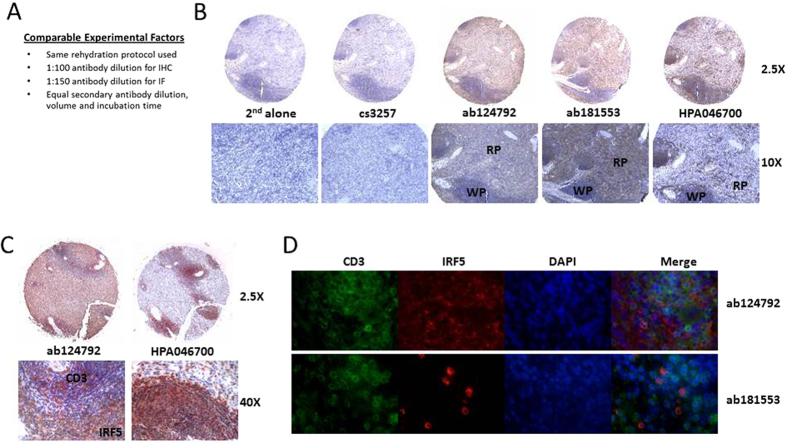
Comparative analysis of IRF5 protein expression in human spleen tissue by IHC and IF microscopy. (**A**) Experimental details for the comparative analysis of α-IRF5 antibody specificity by IHC and IF microscopy. (**B**) Representative core images from IHC analysis of human spleen tissue. IRF5-positive staining is brown, hematoxylin (blue) stains cell nuclei; 2^nd^ alone, staining with secondary antibody only; RP, red pulp; WP, white pulp. (**C**) Same as (**B**) except tissue cores were double-stained with α-IRF5 antibodies (brown) and α-CD3 antibodies (red). (**D**) Representative images from IF analysis of α-IRF5 (red) and α-CD3 (green) co-staining in human spleen tissue. DAPI (blue) stains cell nuclei. Images were taken at 60X magnification.

Given the importance of IRF5 as a potential biomarker for autoimmune disease and cancer, we evaluated the ability of antibodies to detect IRF5 by immunohistochemical (IHC) and immunofluorescence (IF) microscopy in human splenic tissue. Spleen was chosen for comparative analysis of antibody detection since previous data from Northern blot and qRT-PCR analysis of *IRF5* transcript expression showed high levels of IRF5 in this organ^1^. Antibodies cs3257, ab124792 and ab181553 were chosen for testing on formalin-fixed paraffin-embedded (FFPE) spleen tissue as they provided positive detection in others assays (Figs 1, 2, 3). Antibody HPA046700 (Sigma) was also included in the analysis as it was shown to detect high levels of IRF5 in both red and white pulp of spleen by IHC from The Human Protein Atlas (http://www.proteinatlas.org/ENSG00000128604-IRF5/tissue/spleen). All four antibodies were tested by IHC and IF but only ab124792 and ab181553 worked for IF (Fig. 4 and data not shown). Antibody cs3257 was unable to detect IRF5 by IHC or IF as staining was equivalent to background levels detected with secondary antibody alone (Fig. 4B and data not shown). Ab124792 and ab181553 showed positive staining for IRF5, as compared to secondary alone, and the staining pattern was similar between the two antibodies. Both antibodies detected IRF5 in the red and white pulp, yet staining was distinctly higher in the red pulp. HPA046700 also showed positive staining; however, the pattern of staining was distinct from the two Abcam antibodies. Similar to that shown on the Human Protein Atlas website, high levels of IRF5 were detected in both red and white pulp of human spleen, with higher levels detected in the white pulp. White pulp is primarily made up of B and T cells. Given that IRF5 expression is extremely low in normal, healthy T cells, we performed co-staining with anti-CD3 antibodies to delineate antibody specificity. Data from IHC clearly show that ab124792 stained positive for IRF5 (brown) in cells lacking CD3 co-stain (red). However, HPA046700 stained positive for IRF5 in CD3-positive T cells suggesting non-specific binding by this antibody. Further analysis of co-staining by IF confirmed that ab124792 and ab181553 show IRF5-positive staining in cells that lack CD3 co-stain (Fig. 4D). Together, these data support the use of ab124792 or ab181553 for the specific detection of human IRF5 in FFPE samples by IHC or IF.

**Figure 5:**
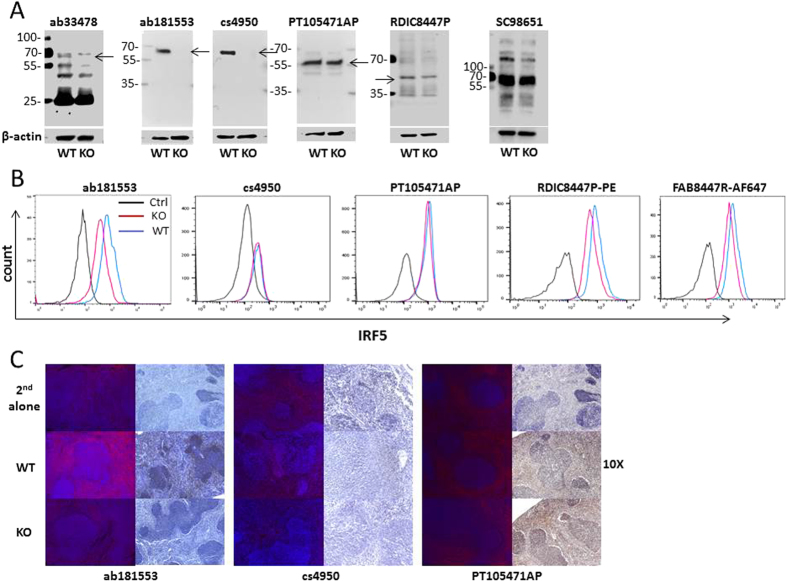
Detection of murine IRF5 by immunoblot, flow cytometry and IHC/IF analysis. (**A**) Immunoblot analysis of endogenous IRF5 protein expression in primary splenocytes from *Irf5*^+/+^ (WT) and *Irf5*^*−/−*^ (KO) mice. 25 μg protein was separated by SDS-PAGE, as described in the Methods section. β-actin levels and apparent molecular weight standards are shown as loading controls and for size comparison, respectively. Arrows indicate detection of an appropriately sized band(s) corresponding to murine IRF5. (**B**) Representative histogram plots from flow cytometry analysis of intracellular IRF5 expression in splenocytes from *Irf5*^+/+^ (WT) and *Irf5*^*−/−*^ (KO) mice. Ctrl, matched isotype antibodies used as non-specific controls. Data are representative of three independent experiments. (**C**) Representative images from IF (left panels) and IHC (right panels) analysis of IRF5 expression in spleen from *Irf5*^+/+^ (WT) and *Irf5*^*−/−*^ (KO) mice. For IF, IRF5-positive staining is red; DAPI is blue. For IHC, IRF5-positive staining is brown; hematoxylin is blue; 2^nd^ alone, staining with secondary antibody only.

Last, we evaluated commercially available antibodies reported by the manufacturer to detect murine IRF5. For this study, spleen from wild-type (WT) and *Irf5*ko (KO) BALB/c mice was used to examine antibody specificity. Six antibodies were tested by immunoblot analysis and two showed highly specific detection of endogenous IRF5 – ab181533 (Abcam) and cs4950 (Cell Signaling); apparent by the single band of correct size in the WT lane and not in the KO lane (Fig. 5A). Ab33478 (Abcam) also detected a band in WT cells that corresponded to the size of IRF5 and was lacking in KO cells, yet numerous other non-specific bands were detected as well. Antibodies PT10547-1-AP (Proteintech), IC8447P (R&D), and SC98651 (Santa Cruz) were unable to detect differences in IRF5 expression between WT and KO splenocytes. Antibodies were then evaluated for their use in flow cytometry using manufacturer’s recommended protocols for fixation, permeabilization and staining. Methods were further optimized in an identical manner by increasing Triton X-100 concentration and incubation time to enhance permeabilization. Data in Fig. 5B was from permeabilization of cells with 0.5% Triton X-100 for 20 min, followed by washing with 0.1% Triton X-100. All un-conjugated antibodies were tested with Alexa Fluor (AF) 488 and 647 secondary antibodies. Neither cs4950 nor PT105471AP were able to detect differential expression between WT and KO splenocytes. We also purchased two recently available pre-conjugated antibodies, PE-conjugated IC8447P (R&D; RDIC8447P-PE) and AF647-conjugated FAB8447R (Novus Biologicals; FAB8447R-AF647), for testing. Although a small shift between WT and KO cells was seen with each pre-conjugated antibody, MFI values were not substantially different between peaks (Fig. 5B). In our hands, only ab181553 was able to detect substantial differences in IRF5 expression between WT and KO splenocytes; MFI for WT was >2-fold higher than KO. Antibodies were then tested for specificity by IF and IHC. Similar to findings from flow cytometry analysis, only ab181553 was able to differentiate IRF5 staining between WT and KO mice; both IF and IHC showed similar specificity. Antibody cs4950 was unable to detect any signal over that seen with secondary antibody alone, while PT105471AP gave positive staining that was not distinct between WT and KO spleen. Thus, ab181553 was the most specific in detecting murine IRF5 cellular expression by all three methods tested.

## Discussion

Antibodies are among the most commonly used tools in the biological sciences to identify and isolate proteins of interest. Unfortunately, as documented in two seminal features in *Nature* last year^40^,^41^, commercial antibodies are “littering the field with false findings”^41^. The current study was an effort to validate commercial antibodies in a transparent manner with the inclusion of appropriate positive and negative controls for analysis. The underlying goal(s) of this study was to identify highly specific antibodies for the detection of human and murine IRF5, and to provide validated methods for testing and use of individual IRF5 antibodies to the scientific community. In the past 5 years, IRF5 has become a molecule of high interest given its important role(s) in regulating inflammation and immunity^7^,^8^,^10^,^19^,^22^,^24^, as well as recent new findings that implicate IRF5 in the regulation of neuropathic pain, obesity, myocardial infarction, allograft rejection, atherosclerosis, and metabolic dysfunction^25^,^26^,^27^,^28^,^42^. The body of research focused on IRF5 expression and function in disease pathogenesis continues to grow and therefore, like many other molecules of interest, will require the continual testing and validation of commercial reagents.

Not surprising to many scientists using antibodies in their research to detect and study molecules of interest, here we report that very few commercial antibodies developed for the detection of IRF5 were capable of detecting IRF5. To the contrary, many commonly used commercial antibodies were unable to accurately and/or specifically measure IRF5 protein expression. A cursory review of the literature indicates very few publications include validated positive and negative controls for protein expression analysis by immunoblot, flow cytometry or microscopy. These types of controls, including molecular weight markers, cell type-specific markers, and knockdown analysis, are essential for the accurate analysis of antibody specificity. In the current study, we identified ab181553 as the most specific antibody for detection of human IRF5 expression by immunoblot analysis (Fig. 1). For IP of human IRF5, NB100-1092, ab181553, and ab124792 were capable, yet ab124792 was the most specific for IRF5 (Fig. 2C–F). A number of antibodies showed positive staining (a shift to the right from isotype control) for human IRF5 by flow cytometry analysis, however, only cs13496, SAB1403991 and ab124792 were able to differentiate IRF5 expression levels between cell lines (Fig. 3). While not shown in the current study, pre-conjugated forms of ab124792 are now available and better at detecting intracellular IRF5 expression differences. Clone 2E3-1A11 used to be the only antibody available for the detection of human IRF5 by IHC and IF microscopy [20,43]. We now have identified two more antibodies that appear to specifically detect human IRF5 in FFPE tissues – ab124792 and ab181553 (Fig. 4). Further testing of HPA046700 is warranted given its current use in The Human Protein Atlas and our finding of IRF5 positive staining in splenic CD3-positive T cells with this antibody (Fig. 4C). The Human Protein Atlas is a public-domain resource used by many scientists for analysis of protein expression differences between normal and diseased cells or tissues. Last, we identified a single antibody that specifically recognizes murine IRF5 by immunoblot analysis, flow cytometry, and IHC/IF – ab181553. As shown in Figs 1 and 4, this antibody was also capable of specifically detecting human IRF5 by immunoblot analysis and IHC/IF.

A cursory review of immunogens used to generate each antibody may provide insight into the development of highly specific 2^nd^ generation α-IRF5 antibodies. For instance, the immunogens used to generate the previously validated rabbit polyclonal cs3257 and mouse monoclonal 2E3-1A11 were a synthetic peptide corresponding to carboxyl terminus residues of human IRF5 (cs3257) and full-length recombinant human IRF5 (2E3-1A11), respectively. Antibodies ab181553 and ab124792 are both rabbit monoclonal antibodies; ab181553 was generated from a recombinant fragment of amino terminal human IRF5 (amino acids 1–200) while the fragment used to generate ab124792 is proprietary. The immunogen for HPA046700 was a short region in the amino terminus of human IRF5 (amino acids 85–134). The amino terminus of IRF family members share significant homology as this is a well-conserved DNA binding domain. Conversely, the carboxyl terminus of IRFs is considered distinct from each other thus providing specificity of function^43^. While results reported herein identify α-IRF5 antibodies that specifically detect IRF5 in multiple assays (Table 1), caution should be used in the evaluation and interpretation of IRF5 expression analysis by other antibodies.

## Methods

### Cell lines and lysates

THP-1 monocytic/macrophages (TIB-202), Ramos B cells (CRL-1596), and Jurkat T cells (TIB-152) were newly purchased from ATCC for this study and grown in RPMI-1640 with 10% FBS. Cell lysates of THP-1 (sc-2238), Ramos B cells (sc-2216), and Jurkat T cells (sc-2204) were purchased from Santa Cruz Biotechnology.

### Mice

*Irf5*^−/−^ mice on a C57Bl/6 background were obtained from T. Taniguchi (University of Tokyo, Tokyo, Japan) and T. Mak (University of Toronto, Toronto, Ontario, Canada)^8^. Wild-type (WT) Balb/c mice were purchased from The Jackson Laboratory (Bar Harbor, ME). *Irf5*^−/−^ mice on a C57Bl/6 background were back-crossed onto the Balb/c background to obtain a generation 10 (F10) cohort of *Irf5*^+*/*+^ and *Irf5*^−/−^ Balb/c littermates, by standard breeding techniques. Mice were genotyped for *Irf5* and the dedicator of cytokinesis 2 (*Dock2*) mutation as previously described^44^,^45^. All *Irf5*^+/+^ and *Irf5*^−/−^ littermates used in this study lacked the *Dock2* mutation45. Littermate *Irf5*^+/+^ mice or WT Balb/c were used as controls. All animal experiments were conducted at the Feinstein Institute for Medical Research (FIMR), Northwell Health, following the guidelines of the Institutional Animal Ethics Committee. Animal ethics was approved by the FIMR Animal Ethics Committee and the experiments were carried out in accordance with the approved guidelines.

### Real-time quantitative polymerase chain reaction (qRT-PCR) analysis

Primers for *IRF5* amplification and thermocycler conditions were previously described^32^. All reactions were performed in the QuantStudio 3 Real-Time PCR System (ThermoFisher Scientific). PCR analyses were done in duplicate, with each set of assays repeated three times. To minimize the effects of unequal quantities of starting RNA and to eliminate potential sources of inconsistency, relative expression levels of each gene was normalized to β-actin using the ∆∆Ct method.

### Immunoprecipitation (IP) and immunoblotting

For IP, Ramos B cells were lysed as previously described in 300 μL of RIPA lysis buffer (10 nM Tris-HCL pH 8.0, 1 mM EDTA, 1% Triton X-100, 0.1% Sodium Deoxycholate, 0.1% SDS, and 140 mM NaCl)^46^,^47^. Lysates (250 μg) were pre-cleared with 75 μL of a 50% agarose protein A/G bead slurry for 1 h before IP with α-IRF5 antibodies (Abcam: #33478, #2932, #124792; Cell Signaling: #3257; Sigma-Aldrich: #WH0003663M1; Novus Biologicals: #NB100-1092, #H00003663-M01). IP was performed overnight at 4 °C on pre-cleared lysates using 4 μg of each α-IRF5 antibody or IgG control. IP samples were washed three times in chilled lysis buffer for 15 min. 50 μL of SDS loading buffer was added to final bead pellet, pellet was boiled, and proteins separated by SDS-PAGE (NuPAGE^®^ Tris-Acetate 8% gel, Life Technologies) followed by transfer onto PVDF. Immunoprecipitated IRF5 was detected by immunoblotting with #H00003663-M01 (Novus Biologicals). For immunoblot analysis, protein lysates (25 μg) were separated by SDS-PAGE (NuPAGE^®^ Tris-Acetate gradient gels 3–8% or 4–12%, Life Technologies) and transferred onto 0.45 μm nitrocellulose membranes (Bio-Rad Laboratories). Membranes were blocked in TBS/0.05% Tween 20 containing 5% BSA for 1 h and incubated overnight at 4 °C with α-IRF5 antibodies (Abcam: #33478, #2932, #124792, #181553; Cell Signaling: #3257, #13496, #4950; Sigma-Aldrich: #SAB1403991; Novus Biologicals: #NB100-1092; Proteintech Group Inc.: #10547-1-AP; R&D Systems: #IC8447P; Santa Cruz: #sc-98651) followed by horse radish peroxidase conjugated secondary antibodies (Cell Signaling: α-rabbit #7074S, α-mouse #7076S; Santa Cruz: donkey α-goat #sc-2020). Membranes were incubated with Clarity™ ECL Western Blotting Substrate (Bio-Rad Laboratories) and chemiluminescence detected with a ChemiDoc™ MP Imaging System (Bio-Rad Laboratories). The PageRuler™ Plus Prestained Protein Ladder (ThermoFisher Scientific) was used for size reference.

### siRNA nucleofection

X 10^6^ Ramos B cells were suspended in 300 μL Amaxa buffer SG (246 μL SG buffer + 54 μL Supplement 1, Lonza: #V4XC-3024). 100 μL of resuspended cells was added to three separate Amaxa cuvettes. 0.5 μL of either ddH_2_O (Mock), 100 μM Scrambled siRNA (Scr) (GE Dharmacon Catalog#: D-001810-10-05), or 100 μM IRF5 targeting siRNA (IRF5KD) (GE Dharmacon Catalog#: L-011706-00-0010) was added to each cuvette. Cells were then nucleofected on the Amaxa 4D Nucleofector using program CA-137. After nucleofection, cells were immediately added to 1 mL of RPMI-1640 (+10% FBS, 1x Glutamine, 1X Non-essential Amino Acids), cultured for 24 h, and then pelleted and re-nucleofected with siRNA as before. Cells were then lysed 24 h after the second nucleofection for immunoblot analysis.

### Flow cytometry

For human cell lines, 2 × 10^6^ cells were fixed overnight at 4 °C in 2% formaldehyde, washed, and permeabilized in 0.1% Triton X-100 as previously described^33^,^43^,^46^,^48^. Fixed cells were blocked with 3% BSA and incubated with α-IRF5 antibodies (Abcam: #33478, #2932, #124792, #181553; Cell Signaling: #3257, #13496; Sigma-Aldrich: #SAB1403991; Novus Biologicals: #NB100-1092), according to manufacturer’s recommendations, for 1 h at room temperature. Fluorochrome-conjugated secondary antibodies (Invitrogen: Alexa Fluor (AF) 488-α-rabbit #A-11034, AF 488-α-mouse #A-11029) were used for detection on a BD LSRFortessa (BD Biosciences). For murine splenocytes, 1 × 10^6^ cells were fixed and permeabilized following manufacturer’s recommended protocols for staining with α-IRF5 antibodies (Abcam: #181553; Cell Signaling: #cs4950; Proteintech: #10547-1-AP; R&D Systems: #IC8447P-PE; Sigma-Aldrich: #FAB8447R-AF647). Protocols were further optimized by fixation in formaldehyde, increasing the concentration of Triton X-100 from 0.1 to 0.5% for permeabilization, washing with Triton X-100, and blocking with TruStain fcX™ (BioLegend #101320) before addition of primary antibodies. The final recommended protocol for detection of intracellular murine IRF5 staining is as follows: fixation in 2% formaldehyde for 15 min at 4 °C, permeabilization with 0.5% Triton X-100 for 20 min at 4 °C, wash with 0.1% Triton X-100, resuspend in 0.1% Triton X-100 for overnight incubation at 4 °C, the following morning resuspend cells in 100 μl of 0.1% Triton X-100 with 2 μl TruStain fcX™ per sample for 20 min at room temperature, followed by addition of manufacturer’s recommended primary antibody concentration, and incubation for 40 min at 4 °C. After washing twice with 0.1% Triton X-100, in the case of unconjugated primary antibodies, cells were labeled with fluorochrome-conjugated secondary antibodies (Invitrogen: #A-10931 APC-anti-rabbit, #A-11034 AF 488-anti-rabbit) and fluorescence detected on a BD LSRFortessa (BD Biosciences).

### Immunohistochemical (IHC) and immunofluorescence (IF) microscopy

Purchased human spleen arrays (US Biomax Inc: #sp241t, #sp481) were deparaffinized according to manufacturer’s instructions. Antigen retrieval was done in Antigen Unmasking Solution (Vector Laboratories: #H-3301) by heating to 95–100 °C for 30 min in a steamer. For IHC, arrays were sequentially blocked with BloXall (Vector Laboratories: #SP-100) for 10 min at room temperature, Avidin/Biotin Blocking Kit (Vector Laboratories: #SP-2001), and 5% normal serum in 0.1% Triton X-100. Slides were incubated overnight at 4 °C with 1:100 dilution of α-IRF5 antibodies (Cell Signaling: #3257; Abcam: #124792, #181553; Sigma-Aldrich: #HPA046700) in serum blocking buffer. Biotin-conjugated secondary antibodies were then applied followed by incubation with avidin-biotin complex (Vector Laboratories: VECTASTAIN Elite ABC Kit #PK-6101). IRF5 staining was visualized with ImmPACT™ DAB Substrate Kit (Vector Laboratories: #SK-4105) and counter-stained with Hematoxylin^20^. For detection of IRF5/CD3 co-staining, after detection of DAB, slides were washed and incubated with 1:100 α-CD3 antibodies (Bio-Rad: #MCA1477T) at 4 °C overnight. CD3 staining was visualized with the VECTASTAIN ABC-AP Kit (Vector Laboratories: #AK-5004) and ImmPACT™ Vector^®^ Red (Vector Laboratories: #SK-5105). For IF, slides were permeabilized with 0.2% Triton X-100 for 20 min at room temperature, washed, and treated with Image-iT™ FX Signal Enhancer (ThermoFisher Scientific: #I36933) for 30 min. Slides were blocked with 4% BSA for 1 h and incubated with a dilution of 1:150 α-IRF5 antibodies and α-CD3 antibodies overnight at 4 °C. After washing, slides were stained with a dilution of 1:1000 secondary antibodies (Jackson ImmunoResearch: AF 488-anti-rat #712-545-153, Cy3-anti-rabbit #711-165-152) for 1 h followed by washing and mounting with VECTASHIELD^®^ HardSet Mounting Media with DAPI (Vector Laboratories: #H-1500). Images were captured on a Zeiss Axiovert 200 M System (Zeiss) at the indicated magnifications. Analysis of murine spleen by IHC and IF was performed in a similar manner with α-IRF5 antibodies (Abcam: #ab181553; Cell Signaling: #4950; Proteintech Group Inc.: #10547-1-AP).

## Additional Information

**How to cite this article**: Li, D. *et al.* Specific detection of interferon regulatory factor 5 (IRF5): A case of antibody inequality. *Sci. Rep.* **6**, 31002; doi: 10.1038/srep31002 (2016).

## Figures and Tables

**Table 1:** IRF5 antibody details.

Company	Catalogue #	Clonality	Species reactivity	Human					Murine			
				WB	IP	Flow	IHC	IF	WB	Flow	IHC	IF
Abcam												
	33478	mouse mono	H/M	−	−	+/−			+/−			
	2932	goat poly	H	−	+/−	−						
	124792	rabbit mono	H	+/−	++	+	++	++				
	181553	rabbit mono	H/M	++	+	+/−	++	++	++	++	++	++
Cell Signaling												
	3257	rabbit poly	H	++	+/−	++	−					
	13496	rabbit mono	H	−		+						
	4950	rabbit poly	M						++	−	−	−
Novus Biologicals												
	NB100-1092	goat poly	H	+/−	+	−						
	H00003663-M01 (clone 2E3-1A11)	mouse mono	H	++	+							
	FAB8447R-AF647	rat mono	M							+/−		
Sigma-Aldrich												
	WH0003663M1	mouse mono	H	++								
	SAB1403991	mouse mono	H	−		+						
	HPA046700	rabbit poly	H				NS					
Proteintech Inc												
	10547-1-AP	rabbit poly	H/M						−	−	−	−
R&D Systems												
	IC8447P	rat mono	M						−	+		
Santa Cruz												
	sc-98651	rabbit poly	M						−			

Abbreviations: Western blot, WB; immunoprecipitation, IP; Flow, flow cytometry; immunohistochemistry, IHC; immunofluorescence, IF; Human, H; mouse, M. ++, antibody worked very well and was specific; +, antibody worked; +/−, antibody was able to detect IRF5 but not specific; NS, signal was detected but not specific for IRF5.

## References

[b1] Barnes B. J., Moore P. A. & Pitha P. M. Virus-specific activation of a novel interferon regulatory factor, IRF-5, results in the induction of distinct interferon alpha genes. J. Biol. Chem. 276, 23382–23390 (2001). 11303025 10.1074/jbc.M101216200

[b2] Barnes B. J., Kellum M. J., Field A. E. & Pitha P. M. Multiple regulatory domains of IRF-5 control activation, cellular localization and induction of chemokines that mediate T-lymphocyte recruitment. Mol. Cell. Biol. 22, 5721–5740 (2002). 12138184 10.1128/MCB.22.16.5721-5740.2002 PMC133975

[b3] Mancl M. E. *et al.* Two distinct promoters regulate the alternative-spliced human Interferon regulatory factor-5 variants: Multiple variants with distinct cell-type specific expression, localization, regulation and function. J. Biol. Chem. 280, 21078–21090 (2005). 15805103 10.1074/jbc.M500543200

[b4] Barnes B. J. *et al.* IRF-5, a novel mediator of cell-cycle arrest and cell death. Cancer Res. 63, 6424–6431 (2003). 14559832

[b5] Barnes B. J. *et al.* Global and distinct targets of IRF-5 and IRF-7 during innate response to viral infection. J. Biol. Chem. 279, 45194–45207 (2004). 15308637 10.1074/jbc.M400726200

[b6] Hu G., Mancl M. E. & Barnes B. J. Signaling through interferon regulatory factor-5 sensitizes p53-deficient tumor to DNA damage-induced apoptosis and cell death. Canc. Res. 65, 7403–7412 (2005). 10.1158/0008-5472.CAN-05-0583 16103093

[b7] Yanai H. *et al.* Role of IFN regulatory factor 5 transcription factor in antiviral immunity and tumor suppression. Proc Natl. Acad. Sci. USA 104, 3402–3407 (2007). 17360658 10.1073/pnas.0611559104 PMC1805533

[b8] Takaoka A. *et al.* Integral role of IRF-5 in the gene induction programme activated by Toll-like receptors. Nature 434, 243–249 (2005). 15665823 10.1038/nature03308

[b9] Schoenemeyer A. *et al.* The interferon regulatory factor, IRF5, is a central mediator of TLR7 signaling. J. Biol. Chem. 280, 17005–17012 (2005). 15695821 10.1074/jbc.M412584200

[b10] Sigurddson S. *et al.* Polymorphisms in the tyrosine kinase 2 and interferon regulatory factor 5 genes are associated with systemic lupus erythematosus. Am. J. Hum. Genet. 76, 528–537 (2005). 15657875 10.1086/428480 PMC1196404

[b11] Graham R. R. *et al.* A common haplotype of interferon regulatory factor 5 (IRF5) regulates splicing and expression and is associated with increased risk of systemic lupus erythematosus. Nat. Genet. 38, 550–555 (2006). 16642019 10.1038/ng1782

[b12] Kozyrev S. V. *et al.* Structural insertion/deletion variation in IRF5 is associated with a risk haplotype and defines the precise IRF5 isoforms expressed in systemic lupus erythematosus. Arthritis Rheum. 56, 1234–1241 (2007). 17393452 10.1002/art.22497

[b13] Sigurdsson S. *et al.* Comprehensive evaluation of the genetic variants of interferon regulatory factor 5 reveals a novel 5bp length polymorphism as strong risk factor for systemic lupus erythematosus. Hum. Mol. Genet. 17, 872–881 (2008). 18063667 10.1093/hmg/ddm359

[b14] Graham R. R. *et al.* Three functional variants of IFN regulatory factor 5 (IRF5) define risk and protective haplotypes for human lupus. Proc. Natl. Acad. Sci. USA 104, 6758–6763 (2007). 17412832 10.1073/pnas.0701266104 PMC1847749

[b15] Rueda B. *et al.* Analysis of IRF5 gene functional polymorphisms in rheumatoid arthritis. Arthritis Rheum. 54, 3815–3819 (2006). 17133578 10.1002/art.22271

[b16] Garcia-Bermudez M. *et al.* Interferon regulatory factor 5 genetic variants are associated with cardiovascular disease in patients with rheumatoid arthritis. Arthritis Res. Ther. 16, R146 (2014). 25011482 10.1186/ar4608 PMC4227041

[b17] Miceli-Richard C. *et al.* The CGGGG insertion/deletion polymorphism of the IRF5 promoter is a strong risk factor for primary Sjögren’s syndrome. Arthritis Rheum. 60, 1991–1997 (2009). 19565491 10.1002/art.24662

[b18] Dideberg V. *et al.* An insertion-deletion polymorphism in the interferon regulatory factor 5 (IRF5) gene confers risk of inflammatory bowel disease. Hum. Mol. Genet. 16, 3008–3016 (2007). 17881657 10.1093/hmg/ddm259

[b19] Eames H. L., Corbin A. L. & Udalova I. A. Interferon regulatory factor 5 in human autoimmunity and murine models of autoimmune disease. Transl. Res. 157, 167–182 (2016). 26207886 10.1016/j.trsl.2015.06.018

[b20] Bi X. *et al.* Loss of interferon regulatory factor 5 (IRF5) expression in human ductal carcinoma correlates with disease stage and contributes to metastasis. Breast Cancer Res. 13, R111 (2011). 22053985 10.1186/bcr3053 PMC3326553

[b21] Fresquet V. *et al.* High-throughput sequencing analysis of the chromosome 7q32 deletion reveals IRF5 as a potential tumour suppressor in splenic marginal-zone lymphoma. Br. J. Haemtol. 158, 712–726 (2012). 10.1111/j.1365-2141.2012.09226.x 22816737

[b22] Krausgruber T. *et al.* IRF5 promotes inflammatory macrophage polarization and TH1-TH17 responses. Nat. Immunol. 12, 231–238 (2011). 21240265 10.1038/ni.1990

[b23] Duffau P. *et al.* Promotion of inflammatory arthritis by interferon regulatory factor 5 in a mouse model. Arthritis Rheumatol. 67, 3146–3157 (2015). 26315890 10.1002/art.39321 PMC4661118

[b24] Weiss M. *et al.* IRF5 controls both acute and chronic inflammation. Proc. Natl. Acad. Sci. USA 112, 11001–11006 (2015). 26283380 10.1073/pnas.1506254112 PMC4568217

[b25] Watkins A. A. *et al.* IRF5 deficiency ameliorates lupus but promotes atherosclerosis and metabolic dysfunction in a mouse model of lupus-associated atherosclerosis. J. Immunol. 194, 1467–1479 (2015). 25595782 10.4049/jimmunol.1402807 PMC4323680

[b26] Dalmas E. *et al.* Irf5 deficiency in macrophages promotes beneficial adipose tissue expansion and insulin sensitivity during obesity. Nat. Med. 21, 610–618 (2015). 25939064 10.1038/nm.3829

[b27] Masuda T. *et al.* Transcription factor IRF5 drives P2X4R+-reactive microglia gating neuropathic pain. Nat. Commun. 5, 3771 (2014). 24818655 10.1038/ncomms4771 PMC4024744

[b28] Courties G. *et al.* *In vivo* silencing of the transcription factor IRF5 reprograms the macrophage phenotype and improves infarct healing. J. Am. Coll. Cardiol. 63, 1556–1566 (2014). 24361318 10.1016/j.jacc.2013.11.023 PMC3992176

[b29] Richez C. *et al.* IFN regulatory factor 5 is required for disease development in the FcgammaRIIB−/−Yaa and FcgammaRIIB−/− mouse models of systemic lupus erythematosus. J. Immunol. 184, 796–806 (2010). 20007534 10.4049/jimmunol.0901748 PMC2858062

[b30] Tada Y. *et al.* Interferon regulatory factor 5 is critical for the development of lupus in MRL/lpr mice. Arthritis Rheum. 63, 738–748 (2011). 21305501 10.1002/art.30183

[b31] Feng D. *et al.* Irf5-deficient mice are protected from pristane-induced lupus via increased Th2 cytokines and altered IgG class switching. Eur. J. Immunol. 42, 1477–1487 (2012). 22678902 10.1002/eji.201141642 PMC3684952

[b32] Feng D. *et al.* Genetic variants and disease-associated factors contribute to enhanced interferon regulatory factor 5 expression in blood cells of patients with systemic lupus erythematosus. Arthritis Rheum. 62, 562–573 (2010). 20112383 10.1002/art.27223 PMC3213692

[b33] Stone R. C. *et al.* Interferon regulatory factor 5 activation in monocytes of systemic lupus erythematosus patients is triggered by circulating autoantigens independent of type I interferons. Arthritis Rheum. 64, 788–798 (2012). 21968701 10.1002/art.33395 PMC3288585

[b34] Stone R. C. *et al.* RNA-Seq for enrichment and analysis of IRF5 transcript expression in SLE. PLoS One 8, e54487 (2013). 23349905 10.1371/journal.pone.0054487 PMC3548774

[b35] Kreher S. *et al.* Mapping of transcription factor motifs in active chromatin identifies IRF5 as key regulator in classical Hodgkin lymphoma. Proc. Natl. Acad. Sci. USA 111, E4513–E4522 (2014). 25288773 10.1073/pnas.1406985111 PMC4210307

[b36] Clark D. N. *et al.* Four promoters of IRF5 respond distinctly to stimuli and are affected by autoimmune-risk polymorphisms. Front. Immunol. 4, 360 (2013). 24223576 10.3389/fimmu.2013.00360 PMC3819785

[b37] Ishikawa C., Senba M. Barnes B. J. & Mori N. Constitutive expression of IRF-5 in HTLV-1-infected T cells. Int. J. Oncol. 47, 361–369 (2015). 26004104 10.3892/ijo.2015.3020

[b38] Kent W. J. *et al.* The human genome browser at UCSC. Genome Res. 12, 996–1006 (2002). 12045153 10.1101/gr.229102 PMC186604

[b39] Rosenbloom K. R. *et al.* ENCODE data in the UCSC Genome Browser: year 5 update. Nucleic Acids Res. 41, D56–D63 (2013). 23193274 10.1093/nar/gks1172 PMC3531152

[b40] Bradbury A. & Plückthun A. Reproducibility: Standardize antibodies used in research. Nature 518, 27–28 (2015). 25652980 10.1038/518027a

[b41] Baker M. Reproducibility crisis: Blame it on the antibodies. Nature 521, 2740276 (2015). 10.1038/521274a 25993940

[b42] Yu X. *et al.* Genetic polymorphism of interferon regulatory factor 5 (IRF5) correlates with allograft acute rejection of liver transplantation. PLoS One 9, e94426 (2014). 24788560 10.1371/journal.pone.0094426 PMC4005731

[b43] Zhao G. N., Jiang D. S. & Li H. Interferon regulatory factors: at the crossroads of immunity, metabolism, and disease. Biochim. Biophys. Acta. 1852, 365–378 (2015). 24807060 10.1016/j.bbadis.2014.04.030

[b44] Purtha W. E., Swiecki M., Colonna M., Diamond M. S. & Bhattacharya D. Spontaneous mutation of the Dock2 gene in Irf5−/− mice complicates interpretation of type I interferon production and antibody responses. Proc. Natl. Acad. Sci. USA 109, E989–904 (2012). 22431588 10.1073/pnas.1118155109 PMC3326475

[b45] Yasuda K. *et al.* Phenotype and function of B cells and dendritic cells from interferon regulatory factor 5-deficient mice with and without a mutation in DOCK2. Int. Immunol. 25, 295–306 (2013). 23291967 10.1093/intimm/dxs114 PMC3631000

[b46] Pimenta E. M. *et al.* IRF5 is a novel regulator of CXCL13 expression in breast cancer that regulates CXCR5(+) B- and T-cell trafficking to tumor-conditioned media. Immunol. Cell. Biol. 93, 486–499 (2015). 25533286 10.1038/icb.2014.110

[b47] Korczeniewska J. & Barnes B. J. The COP9 signalosome interacts with and regulates interferon regulatory factor 5 protein stability. Mol. Cell Biol. 33, 1124–1138 (2013). 23275442 10.1128/MCB.00802-12 PMC3592028

[b48] Yang L., Feng D., Bi X., Stone R. C. & Barnes B. J. Monocytes from *Irf5*^*−/−*^ mice have an intrinsic defect in their response to pristane-induced lupus. J. Immunol. 189, 3741–3750 (2012). 22933628 10.4049/jimmunol.1201162 PMC3454479

